# Small molecules and heat treatments reverse vernalization via epigenetic modification in Arabidopsis

**DOI:** 10.1038/s42003-025-07553-7

**Published:** 2025-01-22

**Authors:** Nana Otsuka, Ryoya Yamaguchi, Hikaru Sawa, Naoya Kadofusa, Nanako Kato, Yasuyuki Nomura, Nobutoshi Yamaguchi, Atsushi J. Nagano, Ayato Sato, Makoto Shirakawa, Toshiro Ito

**Affiliations:** 1 Division of Biological Science, Graduate School of Science and Technology, Nara Institute of Science and Technology (NAIST), Ikoma, Japan; 2 Institute of Transformative Bio-Molecules (WPI-ITbM), Nagoya University, Tokai National Higher Education and Research System, Nagoya, Japan; 3 Faculty of Agriculture, Ryukoku University, Otsu, Japan; 4 Institute for Advanced Biosciences, Keio University, Tsuruoka, Japan

**Keywords:** Plant physiology, Plant development

## Abstract

Monocarpic plants flower only once and then produce seeds. Many monocarpic plants require a cold treatment known as vernalization before they flower. This requirement delays flowering until the plant senses warm temperatures in the spring. Exposure to high temperatures following vernalization causes devernalization, which cancels the vernalized state, inhibiting flowering and promoting vegetative growth. In this study, we screened over 16,000 chemical compounds and identified five small molecules (devernalizers; DVRs) that induce devernalization in *Arabidopsis thaliana* at room temperature without requiring a high-temperature treatment. Treatment with DVRs reactivated the expression of *FLOWERING LOCUS C* (*FLC*), a master repressor of flowering, by reducing the deposition of repressive histone modifications, thereby delaying flowering time. Three of the DVRs identified shared two structures: a hydantoin-like region and a spiro-like carbon. Treatment with DVR06, which has a simple chemical structure containing these domains, delayed flowering time and reduced the deposition of repressive histone modifications at *FLC*. RNA-seq and ChIP-seq analyses revealed both shared and specific transcriptomic and epigenetic effects between DVR06- and heat-induced devernalization. Overall, our extensive chemical screening indicated that hydantoin and spiro are key chemical signatures that reduce repressive histone modifications and promote devernalization in plants.

## Abstract (web summary)

Five compounds that delay flowering time are isolated by high-throughput chemical screening; these small molecules could potentially be used to increase crop and vegetable yields in agricultural fields.

## Introduction

Flowering is an important developmental transition in the plant life cycle. Monocarpic plants flower only once and die after producing seeds. In temperate climates with cold winters, many monocarpic plants require vernalization, a period of low temperature, to induce flowering the following spring^1,2^. This requirement prevents plants from flowering in the autumn, after which cold winter temperatures could kill the plant and prevent seed production. In the monocarpic model plant *Arabidopsis thaliana*, flowering can be induced by two pathways: the vernalization pathway and the autonomous pathway^2–4^. Vernalization is suppressed by the transcriptional activity of the zinc finger protein FRIGIDA (FRI)^5^. Both the vernalization pathway and the autonomous pathway repress the expression of *FLOWERING LOCUS C* (*FLC*), encoding a key repressor of flowering^2,4,6,7^. These pathways counteract the FRI-induced reactivation of *FLC*^5,8^. FLC represses the expression of both the downstream florigen gene *FLOWERING LOCUS T* (*FT*) and the floral inducer gene *SUPPRESSOR OF OVEREXPRESSION OF CONSTANS 1* (*SOC1*)^3,9–11^.

The vernalization pathway inhibits *FLC* transcription by triggering multiple epigenetic modifications. During vernalization, the repressive mark H3K27me3 is deposited at the nucleation region of *FLC* and spreads throughout the *FLC* gene body, whereas the level of the active histone mark H3K4me3 at the *FLC* transcriptional start site is not significantly altered, leading to a bivalent status^12–14^.

The vernalized state following exposure to cold can be effectively cancelled by a high-temperature treatment. This reversal is referred to as devernalization^15,16^, and is a useful agronomic tool for inhibiting vernalization in the field^17–20^. This process results in increased yields of vegetable crops, whose vegetative tissues (e.g., leaves and stems) are consumed and whose value is reduced by flowering. In the model plant Arabidopsis, the H3K27me3 levels decrease and H3K4me3 slightly accumulates at the *FLC* locus during vernalization, leading to a partial recovery of *FLC* expression^21,22^. These observations suggest that the decrease in H3K27me3 levels is a key event during devernalization; however, whether heat treatment globally affects the levels of H3K27me3 at other loci has not been investigated^21,22^. Therefore, the detailed mechanisms of heat-induced devernalization remain largely unknown.

In a previous screening of 3010 compounds, we identified the small molecule DEVERNALIZER 01 (DVR01), which promotes the devernalization of Arabidopsis plants in the absence of high temperatures^23^. The application of DVR01 to vernalized plants induced the upregulation of *FLC* and delayed flowering time; however, DVR01 showed some cell toxicity, and we could not identify which of its chemical structures were critical for its devernalization-inducing activity.

In the current study, to identify non-toxic DVRs and key chemical structures responsible for the induction of devernalization, we performed a blind screening of an additional 13,790 compounds using *FLC* reporter lines. We identified four additional DVRs (DVR02–05) and discovered structures resembling hydantoin and spiro compounds in DVR02, DVR03, and DVR05. Consistent with this finding, DVR06, which has a relatively simple chemical structure resembling hydantoin and spiro compounds, induced devernalization. In addition, DVR06 had almost no toxicity to plants. We also compared the effects of DVR06 and heat treatments on the global transcriptome and the deposition of H3K27me3 using RNA-seq and ChIP-seq, respectively. Our extensive chemical screening revealed that hydantoin and spiro structures are key chemical signatures in molecules (i.e., pharmacophores) that reduce repressive histone modifications and promote devernalization in plants. DVRs have the potential to improve agricultural production by controlling flowering under field conditions.

## Results

### Identification of five DVR compounds in a large-scale screening of a chemical library

**Fig. 1:**
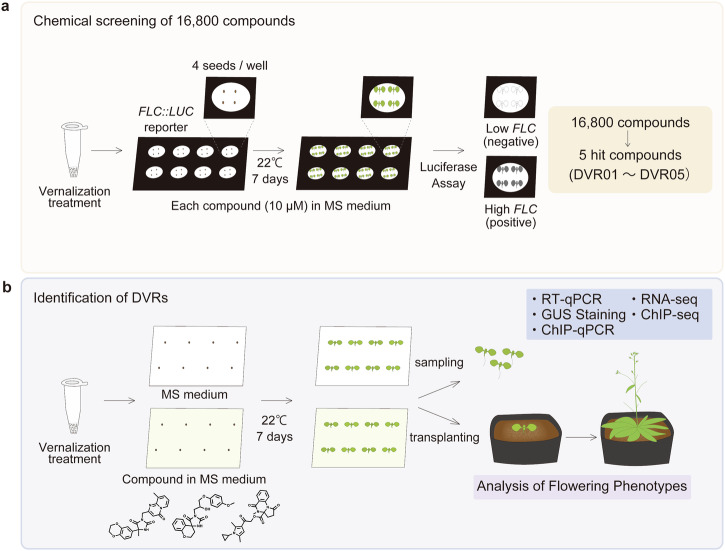
An extensive chemical screening identifies five DVRs. **a** Diagram showing the experimental conditions for the chemical screening to identify DVRs. Hydrated seeds were vernalized in 1.5-mL tubes and sown on cotton balls moistened with the test chemical (10 µM). After one week of cultivation, the plants were sprayed with luciferase substrate (luciferin in a solution containing Triton X-100), and luciferase activity was detected in planta using a CCD camera. **b** Diagram of the experimental strategy for characterizing DVRs. Hydrated seeds were vernalized in 1.5-mL tubes and sown on MS solid medium without (top) or with (bottom) DVRs (10 µM was used, except in the concentration dependency test of DVR06). After one week of cultivation, the plants were collected and subjected to RT-qPCR, GUS staining, ChIP-qPCR, RNA-seq, and ChIP-seq. For the flowering assays, the plants were transferred to soil (vermiculite and Metro-Mix) after one week of cultivation on solid MS medium with DVRs, then grown in the absence of DVRs.

**Fig. 2:**
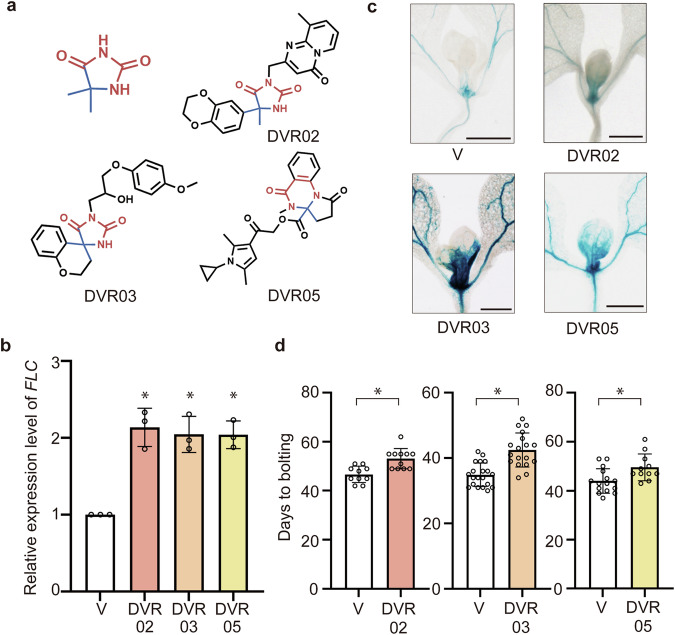
Characterization of the devernalization inducers DVR02, DVR03, and DVR05. **a** Chemical structures of DVR02, DVR03, and DVR05. Hydantoin-containing and spiro-like structures (upper left). Hydantoin-like and spiro-like structures are shown in red and blue, respectively. **b** Expression levels of endogenous *FLC* in vernalized plants (V) and in DVR02-, DVR03-, and DVR05-treated V plants. Error bars represent SD. Small circles represent data from individual plants. *n* = 3. *Significant difference from V plants (*p* < 0.05), as determined using Dunnett’s test. **c** Photographs of cotyledons and leaves of GUS-stained *FLC::GUS* V plants and DVR02-, DVR03-, and DVR05-treated *FLC::GUS* plants. Bars = 500 µm. **d** One week of DVR02, DVR03, and DVR05 treatment delays flowering time in V plants. Left: V: *n* = 10; DVR02: *n* = 11. Middle: V: *n* = 20; DVR03: *n* = 18. Right: V: *n* = 15; DVR05, *n* = 11. Error bars represent SD. Significant differences were determined using a two-tailed Student’s *t*-test (*p < 0.05). V, vernalized plants; DVR, DVR-treated V plants.

To identify DVRs, we used Arabidopsis *FLC::LUC* reporter lines^23^ to screen 16,800 molecules in our Institute of Transformative Bio-Molecules (ITbM) chemical library, an extensive chemical library used for plant-based phenotypic screening^23–37^. In this screen, we identified five DVRs (DVR01–05) (Fig. 1). We previously described research on DVR01^23^ and will describe work characterizing DVR04 in the future. Here, we describe the results of phenotypic, physiological, and biochemical analyses of the three remaining DVR compounds: DVR02, DVR03, and DVR05 (Fig. 2a).

### DVR02, DVR03, and DVR05 induce devernalization in planta

To validate the effects of DVRs on *FLC* expression, we quantified the endogenous expression levels of *FLC* in DVR-treated vernalized Arabidopsis Col *FRI*^*sf-2*^ (hereafter, DVR-treated V plants) compared with the levels in vernalized Col *FRI*^*sf-2*^ (hereafter, V plants) (Fig. 1b). DVR02-, DVR03-, and DVR05-treated V plants showed an approximately two-fold higher level of *FLC* expression compared with the V plants (Fig. 2b**;** *p* < 0.05, Dunnett’s test). The effects of these three DVRs on *FLC* upregulation were slightly weaker than that of DVR01^23^. Next, we used a *FLC::GUS* reporter to examine whether DVRs could activate *FLC* expression in the vascular tissues of V plants, where FLC represses *FT* expression^11^. Compared with the V plants, the DVR02-, DVR03-, and DVR05-treated plants showed increased *FLC* expression in the leaves and vascular tissues (Fig. 2c). When we tested the effects of the DVRs in planta, DVR02, DVR03, and DVR05 delayed bolting relative to the V plants (Fig. 2d**;** *p* < 0.05, two-tailed Student’s *t*-test). Collectively, these results confirm the notion that DVR02, DVR03, and DVR05 are inducers of devernalization in Arabidopsis. Notably, these three heterocyclic compounds share two structures. One is a spiro (DVR03) or spiro-like (DVR02 and DVR05) structure (blue in Fig. 2a), and the other is a hydantoin (DVR02 and DVR03) or hydantoin-like (DVR05) structure (red in Fig. 2a). These results suggest that hydantoin-containing and spiro structures are features of DVRs.

### Small molecules containing hydantoin and spiro structures are sufficient to induce devernalization

**Fig. 3:**
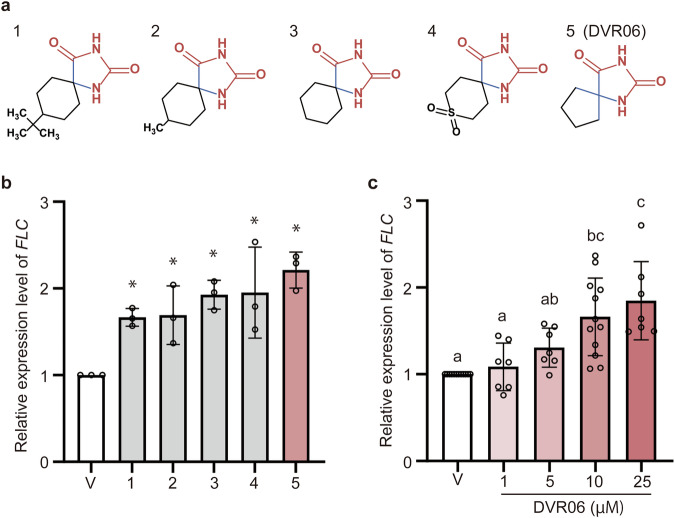
Analogous compounds with hydantoin-containing and spiro structures upregulate the expression of *FLC.* **a** Chemical structures of five compounds with hydantoin-containing and spiro structures. **b** Expression levels of endogenous *FLC* in untreated vernalized plants (V) and V plants treated with the five analogous compounds shown above. *n* = 3. Error bars represent SD. *Significant difference from V plants (*p* < 0.05), as determined using Dunnett’s test. **c**, Expression levels of endogenous *FLC* in V plants and DVR06-treated V plants using 0 (V), 1, 5, 10, or 25 µM DVR06. V: *n* = 12; 1 µM: *n* = 7; 5 µM: *n* = 7; 10 µM: *n* = 12; 25 µM: *n* = 7. Error bars represent SD. Different letters indicate significant differences (one-way ANOVA followed by the Tukey-Kramer test, *p* < 0.05). V, vernalized plants; DVR, DVR-treated V plants.

To examine whether the hydantoin and spiro structures are sufficient to trigger devernalization, we tested five additional small compounds containing both hydantoin and spiro structures (Fig. 3a). All these compounds induced approximately two-fold higher levels of *FLC* expression than the V plants (Fig. 3b**;** *p* < 0.05, Dunnett’s test). By contrast, small compounds that possessed either a hydantoin or a spiro structure alone did not increase *FLC* expression (Fig. S1), suggesting that both structures are required for the upregulation of *FLC*. We selected the simplest of these active molecules, 1,3-diazaspiro[4.4]nonane-2,4-dione, which we named DVR06, and discovered that it triggered the highest level of *FLC* induction (Fig. 3b**;** *p* < 0.05, compared with the V plants using Dunnett’s test). To examine the concentration-dependent effects of DVR06, we treated V plants with DVR06 at concentrations of 1, 5, 10, and 25 µM. Both 10 and 25 µM DVR06 significantly induced the expression of *FLC* compared with the V plants (Fig. 3c**;** *p* < 0.05, Tukey-Kramer test), suggesting that DVR06 regulates *FLC* expression in a concentration-dependent manner.

### DVR06 upregulates *FLC* expression in the leaves of V plants

**Fig. 4:**
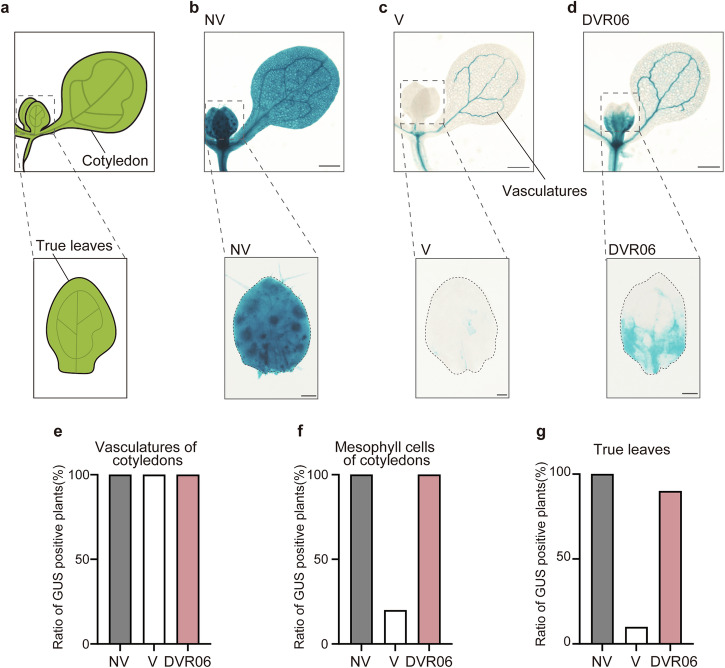
DVR06 upregulates *FLC* expression in the meristematic and vascular tissues of the leaves of V plants. **a** Diagrams of a seedling with cotyledons and true leaves. **b** Photographs of GUS-stained leaves of NV (non-vernalized) *FLC::GUS* plants. **c** Photographs of GUS-stained leaves of V (vernalized) *FLC::GUS* plants. **d** Photographs of GUS-stained leaves of DVR06-treated V *FLC::GUS* plants. Bars = 500 µm (upper) and 100 µm (lower). **e–g** Ratio of GUS-positive plants in different tissues. **e** Vasculature of cotyledons; **f** mesophyll cells of cotyledons; **g** true leaves. NV: *n* = 10; V: *n* = 10; DVR06: *n* = 10. V, vernalized plants; NV, non-vernalized plants; DVR, DVR-treated V plants.

*FLC* is expressed in almost all plant tissues, including aerial parts and roots. The expression of *FLC* in vascular and meristematic tissues in the aerial parts of plants is critical for the repression of *FT*^11^. To examine where DVR06 induces *FLC* expression in intact seedlings, we used the translational fusion line *FLC::GUS* as a reporter and visualized GUS expression in V plants, non-vernalized (NV) plants, and V plants treated with 10 µM DVR06. NV plants showed extremely strong *FLC::GUS* signals throughout the plant because FRI induced the active transcription of *FLC* without any epigenetic repression (Fig. 4a, b). We detected much stronger GUS signals in vascular and meristematic tissues of both cotyledons and true leaves of DVR06-treated V plants than in the V *FLC::GUS* plants (Fig. 4a, c, d). The vasculatures of cotyledons of all plants displayed GUS signals (Fig. 4e); however, while 90% of DVR06-treated V plants had GUS signals in the mesophyll cells of their cotyledons (Fig. 4f) and true leaves including vascular tissues (Fig. 4g), only 10% of V plants showed a GUS signal (DVR06-treated V plants *n* = 10; V plants, *n* = 10). These results indicate that DVR06, like DVR02, DVR03, and DVR05, specifically and effectively induces the reactivation of *FLC* expression in the leaves.

### DVR06 induces devernalization

**Fig. 5:**
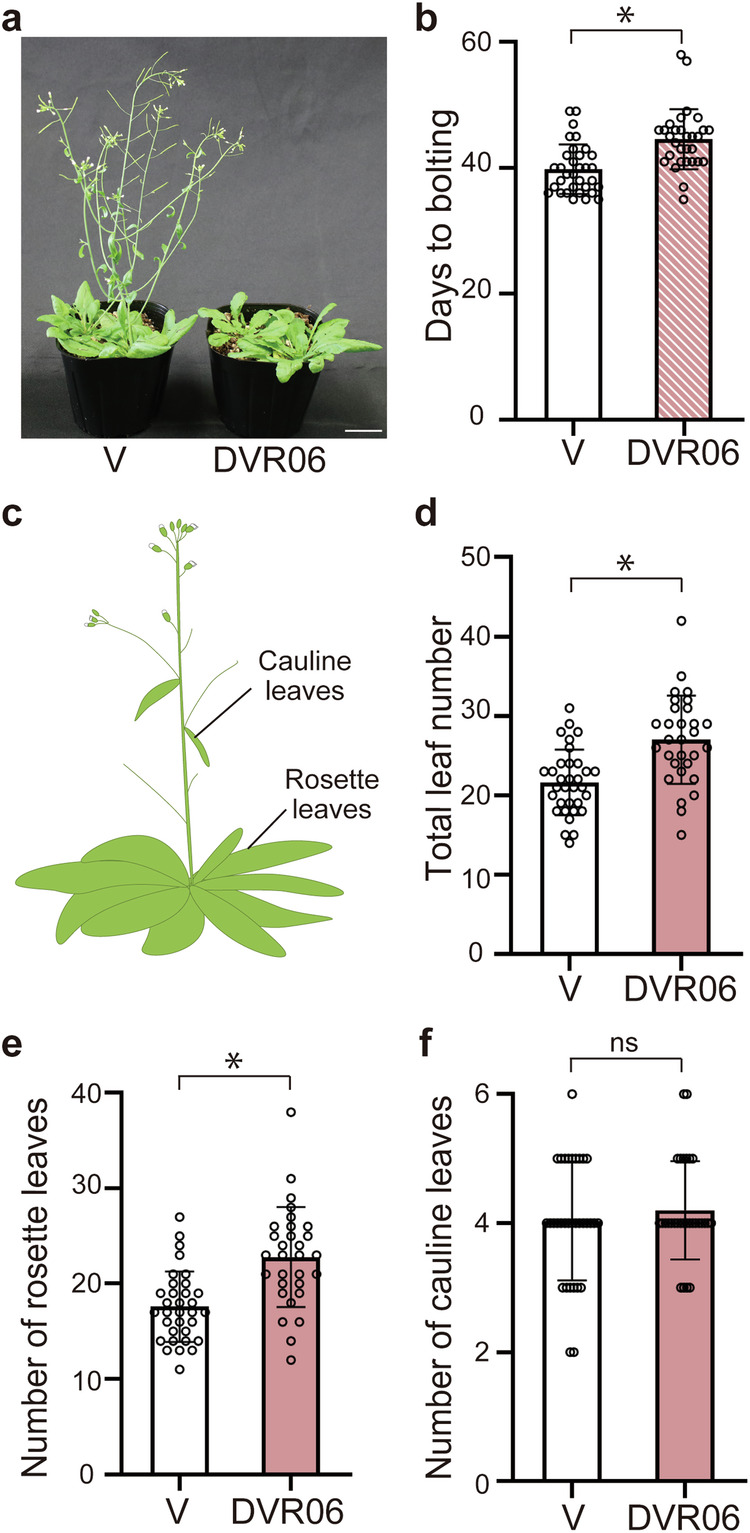
DVR06 induces devernalization. **a** Photograph of 40-day-old plants (left: V plants, right: DVR06-treated V plants). Bars = 3 cm. **b** One week of DVR06 treatment delays flowering time in V plants, as determined by days to bolting (flowering time is defined as the time when the primary stem length reaches 10 cm). V: *n* = 33, DVR06: *n* = 30. Error bars represent SD. *Significant difference, as determined using a two-tailed Student’s *t*-test (*p* < 0.05). **c** Diagram of the two types of leaves (cauline and rosette leaves) that were counted in the flowering time assay. **d–f** One week of DVR06 treatment delays flowering time in V plants, as determined by the number of leaves in flowering plants (flowering time is defined as the time when the primary stem length reaches 10 cm). V: *n* = 33; DVR06: *n* = 30. **d** Total leaf number; **e** number of rosette leaves; **f** number of cauline leaves. Error bars represent SD. *Significant difference, as determined using a two-tailed Student’s *t*-test (*p* < 0.05). V, vernalized plants; DVR, DVR-treated V plants.

Because DVR02, DVR03, and DVR05 delayed the time of bolting relative to the V plants (Fig. 2d), and DVR06 reactivated *FLC* expression (Figs. 3, 4), we examined whether a DVR06 treatment could confer late-flowering phenotypes to V plants. We cultured seedlings for 1 week in a medium containing 10 µM DVR06, transferred them to soil, and counted both the number of days to bolting and the number of rosette and cauline leaves as indicators of flowering time (Fig. 5). As reported previously^23^, NV plants had not bolted by the end of the cultivation period (90 days, *n* = 5; average of 99 leaves at day 90). In contrast to V plants (*n* = 33), DVR06-treated V plants (*n* = 30) showed a late-flowering phenotype (Fig. 5a, b). DVR06-treated V plants bolted five days later than V plants (Fig. 5b; 45 days for DVR06-treated V plants vs. 40 days for V plants; *p* < 0.05) and had increased vegetative growth relative to V plants (Fig. 5c–f; DVR06-treated V plants developed four more leaves than V plants; *p* < 0.05). These results indicate that DVR06 acts as a key chemical signature (i.e., pharmacophore) that promotes devernalization in plants and highlights its potential for regulating flowering time for agricultural purposes.

### DVR06-treated V plants have low levels of H3K27me3 at the *FLC* locus

**Fig. 6:**
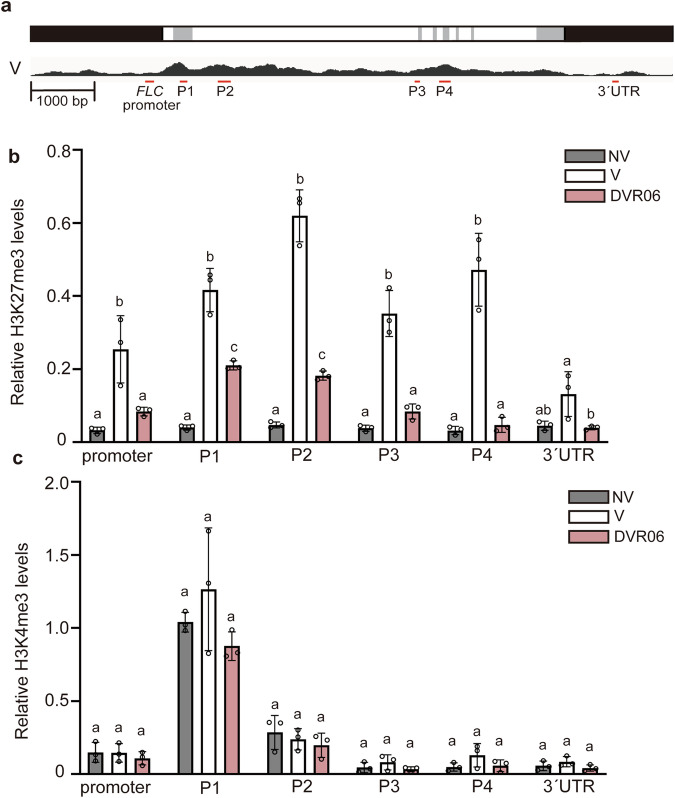
Low levels of H3K27me3 accumulate at the *FLC* locus in DVR06-treated V plants. **a** Sites of PCR amplicons on the *FLC* locus. Upper: a gene model of *FLC* locus. Lower: the pattern of H3K27me3 accumulation on *FLC* locus in vernalized plants (V) based on ChIP-seq data (see Fig. 8). Promoter and 3′-untranslated region (UTR): black; intron: white; exon: gray. Red bars indicate the regions amplified by qPCR. **b**, **c** Accumulation levels of H3K27me3 (b) and H3K4me3 (c) in NV (non-vernalized), V (vernalized), and DVR06-treated V plants. The P1 region is located in the nucleation region. The ratios of ChIP to input DNA (% input) in each region were normalized using the value of % input for the positive control STM. Three independent experiments were performed. Error bars represent SD. Different letters indicate significant differences (*p* < 0.05), as determined using a one-way ANOVA followed by a Tukey-Kramer test. V, vernalized plants; NV, non-vernalized plants; DVR, DVR-treated V plants.

Vernalization triggered the accumulation of the repressive epigenetic mark H3K27me3 at the *FLC* locus (Fig. 6). During cold treatment (4 °C in the dark), the accumulation of H3K27me3 was observed in the first intron of the *FLC* locus, an area known as the nucleation region (P1 in Fig. 6a). This epigenetic mark then spread throughout the genomic region of *FLC* (promoter, P2–P4 in Fig. 6a), except for the 3′-untranslated region (UTR), during further cultivation (22 °C in the light)^13^. We compared the accumulation levels of H3K27me3 throughout the *FLC* genomic region in NV, V, and DVR06-treated V plants using ChIP-qPCR with six different primer sets, which covered the entire *FLC* locus (Fig. 6a). Compared with the NV plants, V plants exhibited much higher levels of H3K27me3 throughout the *FLC* locus, excluding the 3′-UTR. These results are consistent with previous findings^13^. The DVR06-treated V plants accumulated less H3K27me3 at the *FLC* locus than did the V plants (Fig. 6b; *p* < 0.05, Tukey-Kramer test), although the levels at the promoter and the P3 and P4 regions were similar. In the P1 and P2 regions, the H3K27me3 levels in the DVR06-treated V plants were lower than in V plants but slightly higher than in NV plants (Fig. 6b).

Next, we compared the accumulation levels of the active mark H3K4me3 throughout the *FLC* genomic region in the NV, V, and DVR06-treated V plants using ChIP-qPCR (Fig. 6c). As reported previously^12^, H3K4me3 was highly deposited in the P1 and P2 regions of *FLC* in NV and V plants, with similar levels in the DVR06-treated V plants (Fig. 6c). Taken together, these data indicate that DVR06 is a newly characterized compound that confers a late-flowering phenotype to V plants via the reactivation of *FLC* by reducing the levels of H3K27me3 rather than increasing the levels of H3K4me3.

### DVR06- and heat-induced devernalization induce both shared and specific transcriptomic changes

**Fig. 7:**
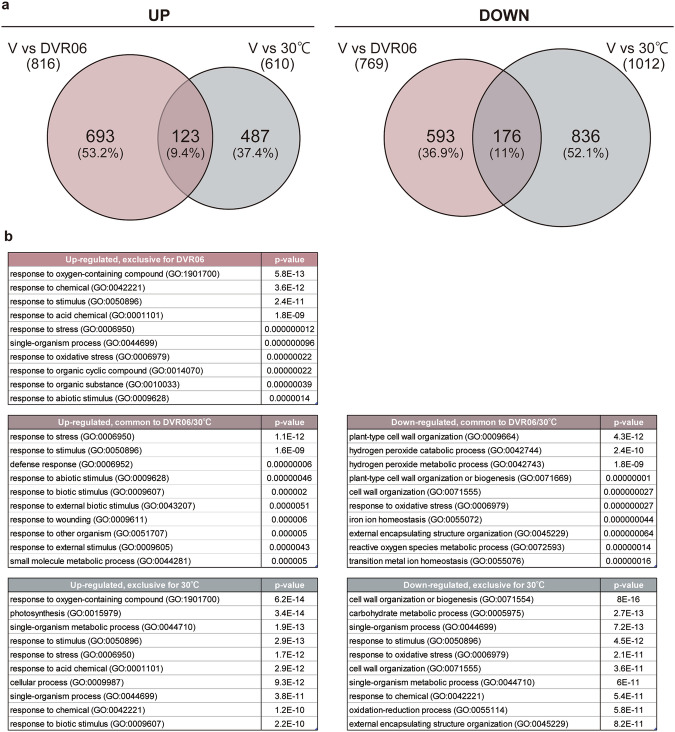
DVR06- and heat-induced devernalization induce both shared and specific transcriptomic changes. **a** Venn diagram of the overlap between transcriptome datasets from Arabidopsis genes induced (UP, left) and suppressed (DOWN, right) by DVR06 (red) or heat treatment (gray), respectively (12 biological replicates, fold change > 1.2 [UP] and FDR < 0.05). **b** Enriched Gene Ontology (GO) categories of overlapping or specific differentially expressed genes (DEGs) following the DVR06/30°C treatments. The top 10 categories are shown in ascending order based on *p*-values. The original data for these DEGs and the GO analysis are shown in Supplementary Data 4. V, vernalized plants; DVR, DVR-treated V plants.

We and another group previously showed that treating vernalized seeds at 30 °C upregulated the expression of *FLC* and induced late-flowering phenotypes^21,22,38^. Importantly, in Arabidopsis, such heat treatments should be performed in the dark (Fig. S2)^38^. Although the DVR treatment was performed in constant light conditions, to comprehensively compare the commonality and specificity between DVR06- and heat-induced devernalization, we performed deep transcriptome sequencing (RNA-seq) on the V plants, DVR06-treated V plants, and heat-treated V plants (Fig. 7). The RNA-seq analysis identified 816 and 610 upregulated genes and 769 and 1012 downregulated genes in the DVR06-treated V plants and heat-treated V plants, respectively (hereafter referred to as DVR06 UP or DOWN and 30 °C UP or DOWN) compared with the V plants (fold change ≥ 1.2 and a false discovery rate [FDR] ≤ 0.05) (Fig. 7a, and Supplementary Data 1–3). There were 123 overlapping UP differentially expressed genes (DEGs; including *FLC*) and 176 DOWN DEGs, respectively, between the two treatments. Enriched Gene Ontology (GO) categories in the UP genes (exclusive to DVR06, common to DVR06/30 °C, and exclusive to 30 °C) included ‘response to chemical’, ‘response to stimulus’, and ‘response to stress’ (Fig. 7b, and Supplementary Data 4). These results suggest that DVR06 and heat treatment shared some DEGs, but they also induced treatment-specific changes in gene expression. We did not exclude the possibility that a subset of transcriptomic difference was caused by the conditions of treatment between DVR06 treatment under the light and heat treatment under the dark.

### The DVR06- and heat-induced devernalization pathways share many target loci

**Fig. 8:**
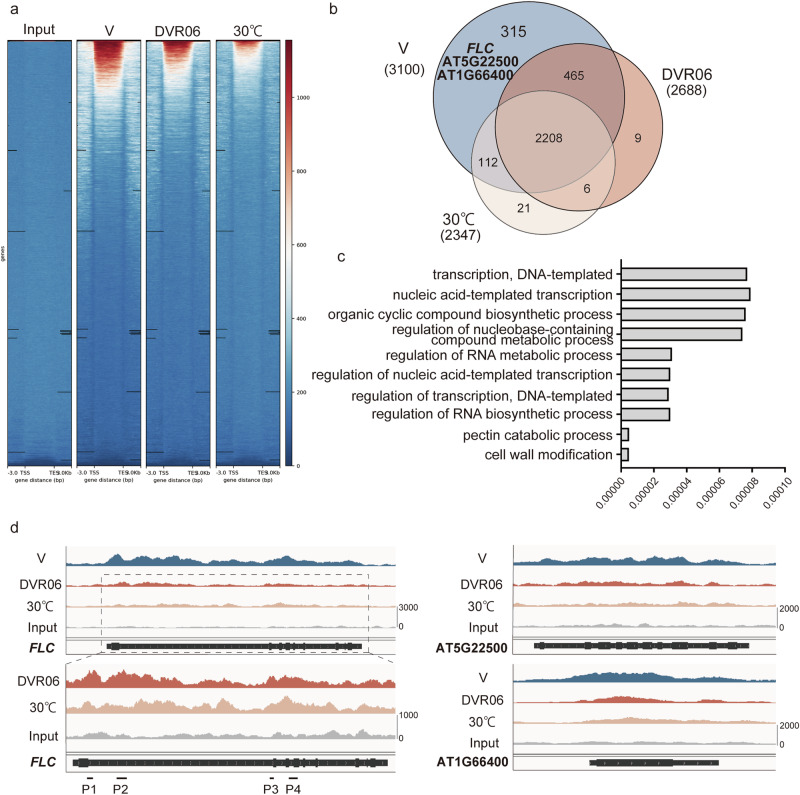
Global changes in H3K27me3 induced by the DVR06 and heat treatments. **a** Metaplot profiles of the H3K27me3 signal intensity around the genes in the input, V, DVR06-treated V (DVR06), and heat-treated V plants (30°C). Panels show the read density heatmaps around each detected peak. **b** Venn diagram of the overlap between the H3K27me3-deposited genes in the V, DVR06-treated V, and heat-treated V plants. Note that H3K27me3 levels in 315 genes were reduced in both DVR06-treated V (DVR06) and heat-treated V plants (30°C). **c** Enriched Gene Ontology (GO) categories of differentially H3K27me3-deposited genes including 315, 465, and 112 genes following the DVR06/30°C treatments. The top 10 categories are shown in ascending order based on *p*-values. The original data for the GO analysis are shown in Supplementary Data 6. **d** Snapshots of Integrative Genomics Viewer (IGV) browser views of V-, DVR06-, and heat-treated samples, as well as input samples at the *FLC*, AT5G22500, and AT1G66400 loci. The gene models are shown as black bars (coding regions) and lines (non-coding regions) at the bottom of each panel. The lower panels show enlarged views of the nucleation regions of *FLC* without the V samples. Note that H3K27me3 peaks were rarely detected at nucleation regions in heat-treated V plants. V, vernalized plants; DVR, DVR-treated V plants; 30°C, heat-treated V plants.

Our transcriptome analysis suggested that the effects of DVR06 and heat were not completely specific to *FLC*. To comprehensively examine the commonality and specificity in the epigenetic modifications induced during DVR06- and heat-induced devernalization, we performed a ChIP-seq analysis of the H3K27me3 marks in V plants, DVR06-treated V plants, and heat-treated V plants (Fig. 8). Compared with the input samples, H3K27me3 was deposited at 3,281 sites (3,100 genes), 2,812 sites (2,688 genes), and 2,452 sites (2,347 genes) in the V plants, DVR06-treated V plants, and heat-treated V plants, respectively (Fig. 8a, b, and Supplementary Data 5). Compared with the V plants, the levels of H3K27me3 at 427 and 780 genes were reduced in the DVR06-treated V plants and heat-treated V plants, respectively. These results suggest that the heat treatment decreased the H3K27me3 levels at more loci in the Arabidopsis genome than did the DVR06 treatment; however, the levels of H3K27me3 were reduced at 315 shared genes between the DVR06- and heat-treated plants, including *FLC* (73.77% of the 427 DVR06-treated V plant DEGs; 40.38% of the 780 heat-treated V plant DEGs), suggesting that a subset of targets was shared between the two treatments. Enriched Gene Ontology (GO) categories in the genes where the levels of H3K27me3 were reduced (exclusive to DVR06 [112], common to DVR06/30 °C [315], and exclusive to 30 °C [465]) included the regulations of DNA (‘transcription, DNA-templated’, ‘nucleic acid-templated transcription’, ‘regulation of nucleic acid-templated transcription’, ‘regulation of transcription, DNA-templated’) and RNA (‘regulation of RNA metabolic process’, ‘regulation of RNA biosynthetic process’, and ‘response to stress’ (Fig. 8c, and Supplementary Data 6). In addition, the heat treatment more strongly reduced the H3K27me3 mark at the *FLC* locus compared with the DVR06 treatment, especially around the nucleation region (Fig. 8d). These observations might explain the stronger effect of heat on *FLC* upregulation compared with DVR06. A similar trend in the changes in H3K27me3 accumulation was observed at other loci (At5g22500 and At1g66400) (Fig. 8d). Overall, consistent with our RNA-seq data, these results suggest that the two different treatments reduced the H3K27me3 levels to different degrees at thousands of loci, many of which were overlapping.

## Discussion

In this study, we identified the devernalizing compounds DVR02–05 during an extensive chemical screening of over 16,000 compounds (Fig. 1). A structural comparison of three DVRs (DVR02, DVR03, and DVR05) revealed the presence of two key structures, a hydantoin-like region and a spiro-like carbon, which may be important for their devernalization properties (Fig. 2a). The observation that DVR05 has a similar activity to DVR02 and DVR03 suggests that the hydantoin-like structure might be a bioisosteric equivalent to the hydantoin structure. In addition, DVR06, which has a simpler structure but contains the hydantoin and spiro structures, exhibited clear activities as an inducer of devernalization: increased *FLC* expression (Figs. 3, 4), delayed flowering time (Fig. 5), and reduced H3K27me3 accumulation at the *FLC* locus (Fig. 6). Small compounds possessing either a hydantoin or a spiro structure did not alter *FLC* expression (Fig. S1), suggesting that both structures are required for the upregulation of *FLC*. Future studies elucidating the three-dimensional structures of binding between DVR06 and its unknown target(s) (for example, proteins or nucleic acids) will shed light on which chemical structure is critical for this activity.

Hydantoin and spiro structures were not found in DVR01, suggesting that other regions of DVR01 induce devernalization, possibly via different molecular mechanisms. We observed that the effects of DVR02, DVR03, DVR05, and DVR06 on the upregulation of *FLC* were slightly weaker than that of DVR01 (Figs. 2, 3)^23^. In addition, DVR01 has some toxicity to leaves^22^, while the DVRs containing hydantoin and spiro structures were non-toxic inducers of devernalization. The lack of toxicity and lower molecular weight of DVR06 are useful characteristics for chemicals used in field cultivation, making this compound the most suitable of these newly identified DVRs for agricultural purposes. More generally, our study indicates that this type of extensive chemical screening enables the identification of key structures for developmental transitions and devernalization in plants, increasing our understanding of the structural diversity of chemicals useful for devernalization.

The *FLC* locus provides a good model for studying epigenetic regulation. Following cold treatment, the deposition of H3K27me3 is initiated at the nucleation region; later, when plants are exposed to light, H3K27me3 spreads throughout the entire *FLC* locus^39^. At the *FLC* nucleation region and a neighboring region, the H3K27me3 levels were lower in DVR06-treated plants than in the NV plants but higher than in the V plants. At other regions, the H3K27me3 levels in DVR06-treated plants were comparable with those in the V plants (Fig. 6). By contrast, the H3K4me3 levels at *FLC* did not change during the vernalization or DVR06 treatments. Taken together, these results suggest that, after vernalization, the nucleation regions on *FLC* were labeled with both H3K27me3 and H3K4me3 (i.e., a bivalent status). Subsequently, the levels of H3K27me3 were reduced by DVR06, resulting in the activation of *FLC*.

To examine the specificity of the effects of DVR06 on H3K27me3 and compare these effects with a heat treatment, we identified up- or downregulated DEGs and gene loci where the levels of H3K27me3 were reduced in DVR06-treated plants. DVR06 reduced the H3K27me3 levels at 2,812 loci throughout the genome, including *FLC*, suggesting that DVR06 is a potential H3K27me3 inhibitor in Arabidopsis. We compared the loci affected by the DVR06 and heat treatments. DVR06 had fewer target loci than the heat treatment, suggesting that the DVR06 treatment has greater specificity. In addition, the heat treatment more strongly reduced the H3K27me3 levels throughout the *FLC* locus compared with DVR06, pointing to a possible trade-off between specificity and activity against this histone modification. Consistent with the reduced H3K27me3 levels, the heat treatment more strongly upregulated the expression of *FLC* and induced devernalization compared with the DVR06 treatment. In the future, perhaps an improved DVR06 molecule could induce devernalization to a level similar to heat.

Hydantoin and spiro structures display a range of biological activities; for example, certain hydantoin derivatives have shown anti-cancer properties^40^, and hydantoin-spiro-derivatives have been identified for their inhibitory activity against placental aldose reductase^41^. Despite these insights, their molecular interactions are not yet fully understood. In this study, we comprehensively analyzed the effects of DVR06 in plants, although it remains unclear how DVR06 reduces the repressive histone marks at specific loci. Identifying the target(s) of DVRs and structurally analyzing the DVR–target complexes should enhance our understanding of how DVRs function in plant cells, allowing us to develop superior DVRs with greater devernalization-inducing activity and higher target specificity.

## Methods

### Plant materials and growth conditions

All *Arabidopsis thaliana* seed stocks used in this study were in the Columbia (Col-0) background. Details regarding *flc-3*^7^, *FRI*^*sf-2*^ *ref*. ^42^, the reporter line *FLC::GUS*^13^, and *FLC::LUC*^23^ were reported previously. Arabidopsis seeds were grown either on cotton balls moistened with liquid Murashige Skoog (MS) medium (SHIOTANI M.S. Co., Ltd.) in 96-well microtiter plates or on MS plates containing 0.5% gellan gum (WAKO). The plates were incubated at 22 °C under constant light conditions (Biotron, LPH-350SP, NK Systems). To examine their flowering phenotypes, plants were cultivated in pots containing vermiculite and Metro-Mix (vermiculite:Metro-Mix = 2:1) (Sun Gro Horticulture).

### Seed vernalization

Seeds were surface sterilized by soaking in 70% ethanol for 5 min, rinsed in 99.9% ethanol, and dried. Approximately 400 seeds were placed in a 1.5-mL sterilized tube in sterile water (approximately 50 µL). The tubes were wrapped in aluminum foil to protect the seeds from light. For vernalization, the tubes were incubated in a refrigerator at 4 °C for four weeks. The vernalized seeds were sown on cotton balls moistened with liquid MS or on MS plates with/without chemicals (10 µM). Vernalized Col *FRI*^*sf-2*^ plants are referred to as V plants.

### Chemical treatments and luciferase assay

A collection of 16,800 molecules in the ITbM chemical library comprising unique, structurally diverse compounds for use in plant-based phenotypic screening was employed in this study. Chemical treatments were carried out as described previously^43^, with minor modifications^23^. Vernalized seeds were sown on cotton balls moistened with the test chemicals (10 µM) in 96-well microtiter plates. For both the negative and positive controls, non-vernalized seeds, vernalized seeds without chemicals, and vernalized seeds with positive control chemicals (DVR01) were sown on the same plate. A total of 80 chemicals were tested in each 96-well microtiter plate. After one week of cultivation, the plants were sprayed with luciferase substrate (1 mM D-Luciferin [Sigma; L9504] in 0.01% Triton X-100). Luciferase activity was detected in planta using an ImageQuant LAS4000 (GE Healthcare) or FUSION Chemiluminescence Imaging System (Vilber Bio Imaging).

### Heat treatment

Vernalized seeds were sown on MS plates. The plates were incubated at 30 °C for six days in the dark, followed by 22°C for seven days under constant light conditions. The plants were frozen in liquid nitrogen and used for RNA extraction and ChIP-seq experiments.

### Reverse-transcription quantitative PCR (RT-qPCR)

After four weeks of vernalization, 10 seeds each were sown on MS plates containing 0.5% gellan gum supplemented with 1, 5, 10, or 25 µM of the DVR being tested. Samples of seven-day-old seedlings from these plates were frozen in liquid nitrogen. An RNeasy Plant Mini kit (Qiagen) was used to extract total RNA, and an RNase-Free DNase Set (Qiagen) was used to eliminate any contaminating genomic DNA in the RNA samples. Reverse-transcription was performed using PrimeScript RT Master Mix (Takara Bio, Japan). A quantitative PCR was performed using a LightCycler 480 system II (384-well; Roche) and a CFX Opus 384 Real-Time PCR System (Bio-Rad Laboratories), as described previously^44^. Arabidopsis *PP2A* (At1g69960) was used as the internal reference^38^. Each experiment was repeated at least three times. The relative expression level of each gene was calculated using the 2^–ΔΔCT^ method^45^. Primers are listed in Supplementary Data 7.

### GUS staining

After four weeks of vernalization, seeds from each DVR treatment were sown on MS plates containing 0.5% gellan gum and 10 µM DVR06. Seven-day-old seedlings expressing the *FLC::GUS* reporter construct were fixed in 90% acetone for 30 min at room temperature and stained overnight with GUS staining solution (0.5 mg/mL X-Gluc [Gold Biotechnology; G1281C5], 0.1 M sodium phosphate buffer, pH 7.0, 10 mM EDTA, 0.5–5.0 mM potassium ferricyanide, 0.5–5.0 mM potassium ferrocyanide, and 0.1% [v/v] Triton X-100) as described previously^46^ The stained plants were cleared overnight in a chloral hydrate solution (chloral hydrate:water:glycerol, 8:2:1 [by volume]) and dehydrated in 60% glycerol overnight. Representative photographs were taken under an AXIO Zoom V16 (Carl Zeiss) microscope.

### Analysis of flowering phenotypes

To document the flowering time and the number of rosette or cauline leaves produced, seeds were vernalized for four weeks then cultured for one week in a medium containing 10 µM DVR06 before being transferred to soil for further cultivation as described above. Flowering time was defined as the time when the primary stem length reached 10 cm, and the number of leaves was counted at this time.

### ChIP-qPCR

ChIP was carried out as described previously^47^. After four weeks of vernalization, four seeds were sown on MS medium containing 0.5% gellan gum and 10 µM DVR06. Seven-day-old seedlings were collected, and 100–300 mg of seedling tissue was fixed in 1% formaldehyde for 15 min. After quenching the formaldehyde with glycine for 5 min, the tissues were frozen in liquid nitrogen and stored at –80 °C until use. For analysis, the tissues were ground to a fine powder with an ice-cold mortar and pestle. Chromatin was isolated from nuclear extracts prepared in nuclear extraction buffer (100 mM MOPS, pH 7.6, 10 mM MgCl_2_, 0.25 M sucrose, 5% Dextran T-40, 2.5% Ficoll 400, 40 mM β-mercaptoethanol, and protease inhibitors). The chromatin was fragmented using a Bioruptor II (TYPE12) (BMBio). After preclearing, the antibodies were added and the mixtures were rotated overnight at 4 °C. An anti-H3K27me3 or an anti-H3K4me3 antibody (ab6002 or ab8580; Abcam; 2 µL per sample) was added. To immunoprecipitate DNA–protein complexes, Dynabeads (Thermo Fisher Scientific) with Protein A or G (Thermo Fisher Scientific) were used. The beads were washed four times with low-salt buffer and 250 mM LiCl buffer, and DNA was eluted from the beads by an overnight incubation at 65 °C to release the cross-links between the protein and the DNA. The resulting DNA was purified using a QIAquick PCR Purification kit (Qiagen) according to the manufacturer’s protocol. DNA was quantified with a CFX Opus 384 (Bio-Rad Laboratories) or a LightCycler 480 II (Roche) using FastStart Essential DNA Green Master Mix (Roche). The ratio of ChIP to input DNA (% input) was determined based on the reaction threshold cycle for each ChIP sample compared with a dilution series of the corresponding input sample. Relative values were normalized using *STM* (AT1G62360) as a positive control locus. Three independent experiments were performed. Primer sequences are listed in Supplementary Data 7.

### RNA-seq

The RNA-seq library was prepared from approximately 500 ng of total RNA per sample, following the Lasy-Seq v1.1 protocol^48^. Library quality was evaluated with a Bioanalyzer (Agilent Technologies). The sequencing was performed using an Illumina NovaSeqX Plus platform, which produced 150-bp paired-end reads. RNA-Seq data preprocessing and quality control were conducted using Trimmomatic v0.33^49^. The processed reads were aligned to the transcript sequences in TAIR10 using Bowtie 1 (version 1.1.1)^50^ and quantified with RSEM (version 1.3.0)^51^. The resulting RSEM output was further analyzed using RNAseqChef^52^ to define the DEGs. edgeR was used for the DEG analysis, with cutoff criteria of a fold change ≥ 1.2 and an FDR ≤ 0.05. The data have been deposited into the DNA Data Bank of Japan (DRA019421; https://ddbj.nig.ac.jp/search/entry/sra-submission/DRA019421).

### ChIP-seq

ChIP-seq was performed as previously described, with slight modifications^53^. Two grams of tissue from vernalized plants, heat-treated plants, and DVR06-treated plants, which were cultivated for seven days under constant light, rapidly frozen in liquid nitrogen, and stored at −80 °C until use. The frozen tissue was thoroughly ground using a mortar and pestle to obtain a fine powder. The chromatin was fixed and isolated using nuclei isolation buffer (10 mM HEPES, 1 M sucrose, 5 mM KCl, 5 mM MgCl_2_, and 5 mM EDTA) containing 1% (w/v) formaldehyde (Thermo Fisher Scientific). The chromatin was fragmented using an ultrasonicator (Covaris M220) and immunoprecipitated using anti-H3K27me3 antibody (ab6002; Abcam; 2 µL per sample) and Dynabeads protein A (Thermo Fisher Scientific) at 4 °C. The validity of the antibodies was confirmed by the suppliers and by immunoblot analysis in the laboratory before use. Following the immunoprecipitation, the DNA was purified using a DNA Cleanup kit (New England Biolabs). The resulting DNA was used as a template to generate a sequencing library using a ThruPLEX DNA-seq kit (Rubicon Genomics) following the manufacturer’s instructions. The immunoprecipitated fraction was analyzed using a NovaSeq 6000 instrument (Illumina). The resulting FASTQ file underwent quality assessment using FastQC (version 0.11.7) (http://www.bioinformatics.babraham.ac.uk/projects/fastqc/), and the raw reads were subjected to trimming using Trimmomatic (version 0.38)^49^. The reads were mapped to the Arabidopsis TAIR10 genome using Bowtie 2 (version 2.3.4.2)^50^. Peak calling was performed with SICER (version 2.0)^54^, while the read counts were calculated using featureCounts (version 1.6.3). The motif analysis was conducted with HOMER (version 4.1) (http://homer.ucsd.edu/homer/). Heatmaps were generated using deepTools (version 3.2.1)^55^, and binding peaks were visualized in the Integrative Genomics Viewer (version 2.8.13)^56^. The data have been deposited into the DNA Data Bank of Japan (DRA019420; https://ddbj.nig.ac.jp/search/entry/sra-submission/DRA019420).

### Illustration

Illustrations in Figs. 1, 4a, and 5c were drawn by using Adobe Illustrator 2023 and 2024.

### Statistics and reproducibility

Statistical analyses were performed using Microsoft Excel, GraphPad Prism 9, and GraphPad Prism 10. Student’s *t*-tests were used to compare two samples. To compare three or more samples, a one-way analysis of variance (ANOVA) was used to test for significant differences. When significant, Dunnett’s test or Tukey-Kramer tests were used for pairwise comparisons. For experiments conducted without statistical analysis, a minimum of three independent biological replicates were performed to confirm reproducibility. The source data behind the graphs in the paper are listed in Supplementary Data 8.

### Reporting summary

Further information on research design is available in the Nature Portfolio Reporting Summary linked to this article.

## Supplementary information

Supplementary Information

Description of Additional Supplementary Files

Supplementary Data 1-8

Reporting summary

## Data Availability

Sequence data from this study can be found in the GenBank/EMBL data libraries under the following accession numbers: *FLC* (At5g10140) and *STM* (At1g62360). RNA-seq data have been deposited into the DNA Data Bank of Japan (DRA019421; https://ddbj.nig.ac.jp/search/entry/sra-submission/DRA019421). Source data of RNA-seq can be found in Supplementary Data 1-4. ChIP-seq data have been deposited into the DNA Data Bank of Japan (DRA019420; https://ddbj.nig.ac.jp/search/entry/sra-submission/DRA019420). Source data of ChIP-seq can be found in Supplementary Data 5 and 6.
