# Supplementary material for: Small molecules and heat treatments reverse vernalization via epigenetic modification in Arabidopsis
Source: Commun Biol. 2025 Jan 22;8:108. doi: 10.1038/s42003-025-07553-7 (PMC11754793; doi:10.1038/s42003-025-07553-7)
Supplement: Supplementary file 1 — Supplementary Information [file 42003_2025_7553_MOESM1_ESM.pdf]

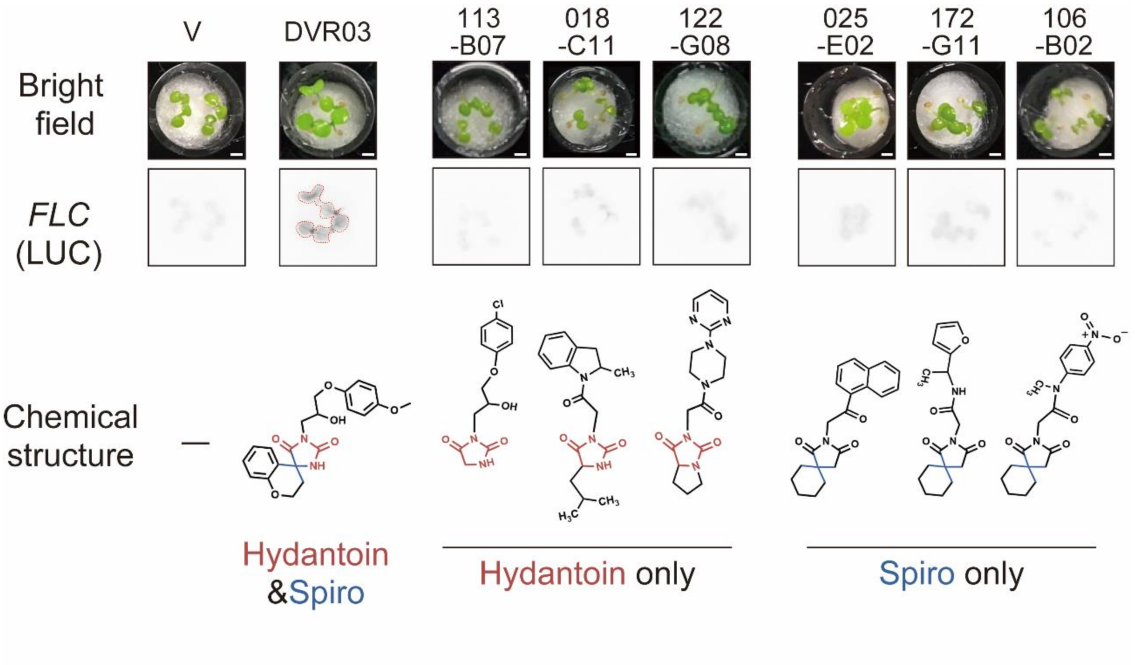

Fig. S1. Both hydantoin and spiro are required for the DVR-mediated activation of *FLC*.

Luciferase activities of *FLC::LUC* in vernalized plants (V) and chemically treated vernalized plants. In the bright-field images (upper), three to four seedlings were grown in each well. In the lower images, black indicates the expression of *FLC*. Bars = 1 mm *FLC* was highly expressed in the DVR03-treated seedlings. Note that compounds containing either hydantoin or spiro alone did not upregulate *FLC*.

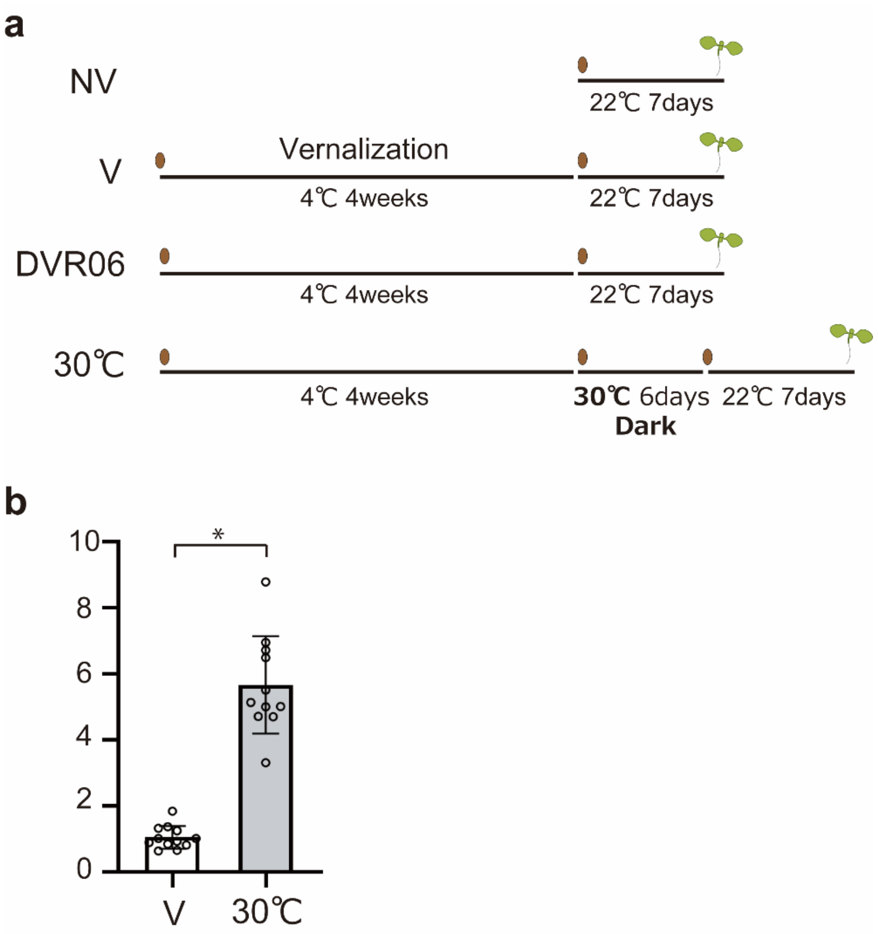

Fig. S2. Heat-induced devernalization.

a, Diagram showing the experimental conditions for chemical and heat treatments. Note that heat treatment was performed in the dark for six days. More than seven days of heat treatment dramatically reduced the germination ratio.

b, Expression levels of endogenous *FLC* in vernalized plants (V; n = 12) and in heat-treated V plants (30°C; n = 11). Error bars represent SD. Small circles represent data from individual plants. *Significant difference from the V plants ($p < 0.05$), determined using Student's t-test.
