# Supplementary material for: Small molecules and heat treatments reverse vernalization via epigenetic modification in Arabidopsis
Source: Commun Biol. 2025 Jan 22;8:108. doi: 10.1038/s42003-025-07553-7 (PMC11754793; doi:10.1038/s42003-025-07553-7)
Supplement: Supplementary file 2 — Description of Additional Supplementary Files [file 42003_2025_7553_MOESM2_ESM.pdf]

Description of Additional Supplementary Files

File name: Supplementary Data 1
Description: Differentially expressed genes between the vernalized (V) and DVR06-treated V plants.

File name: Supplementary Data 2
Description: Differentially expressed genes between the vernalized (V) and heat-treated V plants.

File name: Supplementary Data 3
Description: Comparison of the differentially expressed genes (DEGs) between the DVR06- and heat-treated V plants.

File name: Supplementary Data 4
Description: Results of the Gene Ontology (GO) analysis of RNA-seq.

File name: Supplementary Data 5
Description: H3K27me3 accumulation in the vernalized (V), DVR06-treated, and heat-treated V plants

File name: Supplementary Data 6
Description: Results of the Gene Ontology (GO) analysis of ChIP-seq.

File name: Supplementary Data 7
Description: Primers used in this study.

File name: Supplementary Data 8
Description: The source data behind the graphs in the paper
