# Supplementary material for: Small molecules and heat treatments reverse vernalization via epigenetic modification in Arabidopsis
Source: Commun Biol. 2025 Jan 22;8:108. doi: 10.1038/s42003-025-07553-7 (PMC11754793; doi:10.1038/s42003-025-07553-7)
Supplement: Supplementary file 4 — Reporting summary [file 42003_2025_7553_MOESM4_ESM.pdf]

nature portfolio

Corresponding author(s): SHIRAKAWA, Makoto and ITO, Toshiro.

Last updated by author(s): Dec 17, 2024

Reporting Summary

Nature Portfolio wishes to improve the reproducibility of the work that we publish. This form provides structure for consistency and transparency in reporting. For further information on Nature Portfolio policies, see our Editorial Policies and the Editorial Policy Checklist.

Statistics

For all statistical analyses, confirm that the following items are present in the figure legend, table legend, main text, or Methods section.

n/a	Confirmed	
☐	☒	The exact sample size (*n*) for each experimental group/condition, given as a discrete number and unit of measurement
☐	☒	A statement on whether measurements were taken from distinct samples or whether the same sample was measured repeatedly
☐	☒	The statistical test(s) used AND whether they are one- or two-sided<br>*Only common tests should be described solely by name; describe more complex techniques in the Methods section.*
☒	☐	A description of all covariates tested
☐	☒	A description of any assumptions or corrections, such as tests of normality and adjustment for multiple comparisons
☐	☒	A full description of the statistical parameters including central tendency (e.g. means) or other basic estimates (e.g. regression coefficient) AND variation (e.g. standard deviation) or associated estimates of uncertainty (e.g. confidence intervals)
☐	☒	For null hypothesis testing, the test statistic (e.g. *F*, *t*, *r*) with confidence intervals, effect sizes, degrees of freedom and *P* value noted<br>*Give P values as exact values whenever suitable.*
☒	☐	For Bayesian analysis, information on the choice of priors and Markov chain Monte Carlo settings
☒	☐	For hierarchical and complex designs, identification of the appropriate level for tests and full reporting of outcomes
☒	☐	Estimates of effect sizes (e.g. Cohen's *d*, Pearson's *r*), indicating how they were calculated

Our web collection on statistics for biologists contains articles on many of the points above.

Software and code

Policy information about availability of computer code

Data collection	All software used (including the version and parameter, when not default) is indicated in the Methods section.
Data analysis	All software used (including the version and parameter, when not default) is indicated in the Methods section.

For manuscripts utilizing custom algorithms or software that are central to the research but not yet described in published literature, software must be made available to editors and reviewers. We strongly encourage code deposition in a community repository (e.g. GitHub). See the Nature Portfolio guidelines for submitting code & software for further information.

Data

Policy information about availability of data

All manuscripts must include a data availability statement. This statement should provide the following information, where applicable:

- Accession codes, unique identifiers, or web links for publicly available datasets
- A description of any restrictions on data availability
- For clinical datasets or third party data, please ensure that the statement adheres to our policy

Data supporting the findings of this work are available within the paper (and its supplementary information files). The datasets, plant materials, and chemicals are available from corresponding authors upon request.

Research involving human participants, their data, or biological material

Policy information about studies with human participants or human data. See also policy information about sex, gender (identity/presentation), and sexual orientation and race, ethnicity and racism.

Reporting on sex and gender	n/a
Reporting on race, ethnicity, or other socially relevant groupings	n/a
Population characteristics	n/a
Recruitment	n/a
Ethics oversight	n/a

Note that full information on the approval of the study protocol must also be provided in the manuscript.

Field-specific reporting

Please select the one below that is the best fit for your research. If you are not sure, read the appropriate sections before making your selection.

☒ Life sciences ☐ Behavioural & social sciences ☐ Ecological, evolutionary & environmental sciences

For a reference copy of the document with all sections, see nature.com/documents/nr-reporting-summary-flat.pdf

Life sciences study design

All studies must disclose on these points even when the disclosure is negative.

Sample size	Sample sizes were determined based on prior experience and typical standards in the field. For statistical tests (i.e. Student's t-test, Tukey-Kramer test), at least 3 biological replicates were included to ensure enough sample size.
Data exclusions	Only experimental group and control group were included in the analyses. Any plants without these traits were excluded by phenotyping and/or genotyping.
Replication	All experiments were repeated at least three times, and attempts at replication were successful.
Randomization	Random selection was not conducted. Plants in either experimental group or control group were included in the study. These two groups were distinct from each other; experimental group was compared with control group for phenotyping/expression.
Blinding	Blinding was not applied since experimental group and control group were distinct from each other.

Reporting for specific materials, systems and methods

We require information from authors about some types of materials, experimental systems and methods used in many studies. Here, indicate whether each material, system or method listed is relevant to your study. If you are not sure if a list item applies to your research, read the appropriate section before selecting a response.

Materials & experimental systems

n/a	Involved in the study	
☐	☒	Antibodies
☒	☐	Eukaryotic cell lines
☒	☐	Palaeontology and archaeology
☒	☐	Animals and other organisms
☒	☐	Clinical data
☒	☐	Dual use research of concern
☐	☒	Plants

Methods

n/a	Involved in the study	
☐	☒	ChIP-seq
☒	☐	Flow cytometry
☒	☐	MRI-based neuroimaging

Antibodies

Antibodies used	anti-H3K27me3 (ab6002; abcam)

Antibodies used	anti-H3K4me3 (ab8580; abcam)
Validation	The following antibodies were validated by the suppliers.<br>https://www.abcam.co.jp/products/primary-antibodies/histone-h3-tri-methyl-k27-antibody-mabcam-6002-chip-grade-ab6002.html<br>https://www.abcam.com/en-us/products/primary-antibodies/histone-h3-tri-methyl-k4-antibody-chip-grade-ab8580

Dual use research of concern

Policy information about dual use research of concern

Hazards

Could the accidental, deliberate or reckless misuse of agents or technologies generated in the work, or the application of information presented in the manuscript, pose a threat to:

No	Yes	
☒	☐	Public health
☒	☐	National security
☒	☐	Crops and/or livestock
☒	☐	Ecosystems
☒	☐	Any other significant area

Experiments of concern

Does the work involve any of these experiments of concern:

No	Yes	
☒	☐	Demonstrate how to render a vaccine ineffective
☒	☐	Confer resistance to therapeutically useful antibiotics or antiviral agents
☒	☐	Enhance the virulence of a pathogen or render a nonpathogen virulent
☒	☐	Increase transmissibility of a pathogen
☒	☐	Alter the host range of a pathogen
☒	☐	Enable evasion of diagnostic/detection modalities
☒	☐	Enable the weaponization of a biological agent or toxin
☒	☐	Any other potentially harmful combination of experiments and agents

Plants

Seed stocks	All seed stocks used is indicated in the Methods section.
Novel plant genotypes	All seed stocks used is indicated in the Methods section.
Authentication	All seed used in this study was reported previously. References were cited in the Method Section.

ChIP-seq

Data deposition

☒ Confirm that both raw and final processed data have been deposited in a public database such as GEO.

☒ Confirm that you have deposited or provided access to graph files (e.g. BED files) for the called peaks.

Data access links<br>*May remain private before publication.*	The data was uploaded onto DDBJ website (DRR609305-DRR609308)<br>https://www.ddbj.nig.ac.jp/index.html
Files in database submission	DRR609305-DRR609308

Genome browser session
(e.g. UCSC)

No longer applicable

Methodology

Replicates

Replicates agreed well. Data was further confirmed by ChIP-qPCR

Sequencing depth

Sample Name Total reads Trimmed Reads Mapped reads Mapping rate

Antibodies

anti-H3K27me3 (ab6002; abcam)
V_input 42447202 25994822 23578208 90.70%
V_H3K27me3_1 43174986 29475920 27821575 94.39%
DVR6_H3K27me3_1 42834914 28973516 25659775 88.56%
HDV_HeK27me3_2 43541644 30937744 30508723 98.61%

Peak calling parameters

Peak calling is described in the methods.

Data quality

Peak attributes are described in the methods.

Software

Software used is described in the methods.
